# Tofacitinib as an Adjunct Immunomodulator for Treatment of a Patient with Severe COVID-19: A Case Report

**DOI:** 10.31729/jnma.6680

**Published:** 2021-06-30

**Authors:** Gentle Sunder Shrestha, Bidesh Bista, Ashesh Dhungana, Nimesh Poudel, Shraddha Bhattarai, Manjit Shrestha, Sandip Bhandari, Binit Vaidya

**Affiliations:** 1 Department of Anaesthesiology, Tribhuvan University Teaching Hospital, Kathmandu; 2 Civil Service Hospital of Nepal, Kathmandu; 3 Department of Internal Medicine, National Academy of Medical Sciences, Bir Hospital, Kathmandu; 4 Kathmandu Medical College and Teaching Hospital, Kathmandu; 5 Alka Hospital Pvt. Ltd, Lalitpur; 6 Shahid Gangalal National Heart Centre, Kathmandu; 7 National Center for Rheumatic Diseases, Kathmandu, Nepal

**Keywords:** *COVID-19*, *dexamethasone*, *immunomodulation*

## Abstract

Severe coronavirus disease 2019 can be associated with progressive respiratory failure. In addition to respiratory support and other supportive care, use of corticosteroids has shown to improve outcome. Despite the use of steroids, a significant proportion of patients progressively worsen. Adjunct immunomodulators have been studied in addition to steroids in these patients. Here we present a successful use of tofacitinib, a Janus Kinase inhibitor, in conjunction with dexamethasone for a patient with rapid worsening of respiratory status and with high level of serum inflammatory biomarkers.

## INTRODUCTION

Patients with severe coronavirus disease 2019 (COVID-19) may develop progressive respiratory failure and acute respiratory distress syndrome (ARDS).^1^ The use of dexamethasone in patients requiring oxygen supplementation and respiratory support is associated with better outcome. However, mortality remains high in these patients.^2^ Various immunomodulatory agents like tocilizumab and baricitinib have been studied in patients with severe COVID-19.^3,4^ When used in selective patient population and in conjunction with corticosteroids, these immunomodulatory agents may be beneficial.^5^ Here we present a case who was treated with tofacitinib and with good clinical response. The agent was used together with other standard of care management.

## CASE REPORT

**Figure 1:**
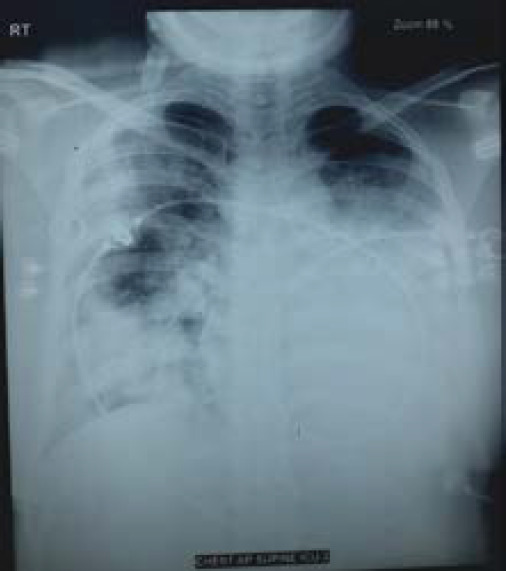
Chest X ray showing bilateral pulmonary infiltrates, predominantly involving left lower zone.

**Figure 2:**
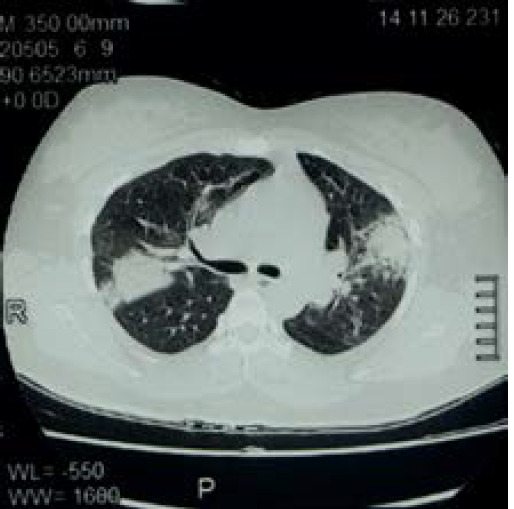
High resolution CT of chest showing bilateral peripheral and peribronchial ground glass opacities with consolidation.

A 31-years-old lady presented with the history of fever since 4 days, dry cough since 3 days and shortness of breath since 1 day. She was a known case of hypothyroidism on thyroxine. She required oxygen supplementation through nasal cannula at 3 litres/minute to maintain peripheral oxygen saturation (SPO_2_) over 92%. She was tachypnoic with respiratory rate of around 25/minute. RT-PCR for SARS-CoV-2 was positive. Chest X ray revealed bilateral pulmonary infiltrates, predominantly involving left lower zone (Figure 1). High resolution CT of chest revealed bilateral peripheral and peribronchial ground glass opacities with consolidation. There was no pleural effusion (Figure 2).

Her total and differential WBC count, haemoglobin level, platelet count, electrolytes, serum creatinine and liver functions tests were within normal limits. Her blood pressure, pulse rate and urine output were normal. She was treated with injectable dexamethasone 6mg daily, enoxaparin 60 mg subcutaneously daily, injectable remdesivir 200mg on day one followed by 100mg daily for four days, injectable paracetamol 1gm 6 hourly for fever, injectable ceftriaxone 1gm twice daily and oral levocetirizine 5 mg daily for dry cough.

**Figure 3:**
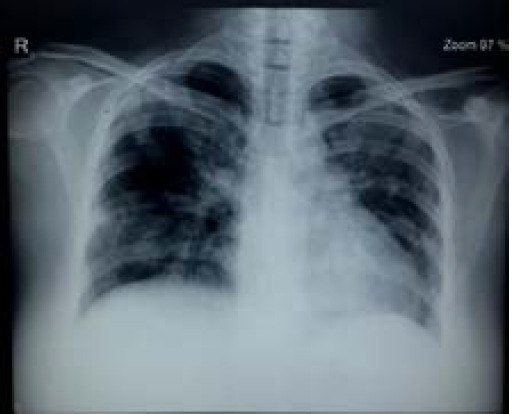
Chest X ray done on day 11 showing increased aeration of bilateral lung fields and significant decrease in pulmonary infiltrates compared to previous chest X ray.

Over the next 24 hours following hospital admission, her respiratory status progressively worsened with increased oxygen requirement and progressive tachypnoea. Oxygen was supplemented with face mask and awake proning was attempted. Due to failure to maintain oxygenation, she was put on non-invasive ventilation with the setting of 15:10 with oxygen flow of 10 litres/minute. Her respiratory rate was 30/ minute. Arterial blood gas (ABG) analysis with these settings revealed normal pH, PCO_2_ of 32 mm Hg, PO_2_ of 70 mm Hg, normal bicarbonate level and normal lactate values. Her serum markers of inflammation were raised, with C-Reactive protein (CRP) level of 88 mg/L, serum ferritin level of 940 ng/ml and LDH level of 1051 U/L. Her D-dimer level was 0.23 ng/dL. Serum procalcitonin level was less than 0.1 ng/ml and her WBC counts were normal. There were no new signs of organ dysfunction other than worsening of respiratory status. Oral tofacitinib 10 mg twice daily was initiated and respiratory support was continued with noninvasive ventilation with close monitoring for possible worsening of respiratory status. Her status remained the same for next 48 hours with no further worsening and no new organ dysfunctions. On day 3 following initiation of tofacitinib, NIV support was progressively decreased upto 10:6. Her ABG parameters were normal and her SPO_2_ was maintained with these settings. CRP level was 51 mg/L. On day 4, she was put on face mask with O_2_ at 6 liters/minute. She could maintain SPO_2_ of 94%. On day 5, she was put on nasal prongs with O_2_ at 3 liters/minute, with which she maintained SPO_2_ of 92%. Bedside ambulation was started. On day 7 she was shifted to ward. Dexamethasone, ceftriaxone and tofacitinib were administered for a total of 10 days. Chest X ray showed progressive improvements (Figure 3).

On day 12 of admission, she could maintain oxygenation with oxygen through nasal prongs at 1 litre/minute. Her CRP level was 12 mg/L, ferritin level was 650 ng/ml and procalcitonin level was 0.04 ng/ml. She was discharged home on day 13 of admission.

## DISCUSSION

Patients with severe COVID-19 who develop ARDS are associated with high mortality. Inflammation, with increased level of inflammatory biomarkers, pulmonary vascular endothelialitis, thrombosis and angiogenesis have been proposed to be the possible pathophysiological basis of pulmonary involvement.^6^ Systemic corticosteroids, anticoagulants and antiviral agents like remdesivir have been widely used for treatment of severe COVID-19, together with respiratory support measures.^1^ Immunomodulation with the use of dexamethasone in hospitalized patients requiring oxygen supplementation of mechanical ventilation is associated with reduced mortality.^2^ However, a significant proportion of patients worsen despite being treated with corticosteroids and anticoagulants.

In patients with severe COVID-19, elevated biomarkers of inflammation is associated with increased disease severity and poor outcome.^7^ Various adjunct immunomodulator agents have been investigated to explore potential benefit in patients with severe COVID-19. Tocilizumab, the interleukin-6 antagonist has been shown to be possibly associated with decreased mortality and reduced need for mechanical ventilation.^3^ The benefit is likely when used in conjunction with corticosteroids, and when used early on during rapid clinical deterioration and those patients with raise biomarkers of inflammation.^5^ Baricitinib, an oral Janus kinase (JAK) 1 and 2, when used together with remdesivir was associated with accelerated clinical improvement in patients requiring oxygen supplementation or on non-invasive ventilation. Baricitinb causes inhibition of expression of various cytokines known to be elevated in severe COVID-19, including interleukin-2, interleukin-6, interleukin-10, interferon-γ and granulocyte-macrophage colony stimulating factor.^4^ However, the role of baricitinib when used together with corticosteroids is not well studied. Also, the use of agents like tocilizumab and baricitinib is largely limited due to cost constraints and limited availability of the agents. The cost for the 400mg dose of tocilizumab is Nepalese rupees 60,000 where as the cost of the full course of tofacitinib is around rupees 4,000.

Tofacitinib, a non-specific JAK inhibitor inhibits all four JAK receptors and suppresses the production of around 10 cytokines. The use of Tofacitinib for patients with moderate to severe ulcerative colitis in phase II and phase III trials showed promising results. Food and Drug Administration (FDA) has approved it for induction and maintenance or remission in these patients. For induction, Tofacitinib is administered 10 mg twice a day for 8 weeks period. The common side effects were headache and nasopharyngitis. Infection requiring parenteral antibiotics and hospitalization were rarely observed.^8^ In a single center retrospective observational study, addition of Tofacitinib to dexamethasone was associated with improved survival. Patients who were hypoxemic and with increased markers of inflammation, with CRP more than 50 mg/L or ferritin more than 500 ng/ml were administered Tofacitinib. However, the study protocol changed with the evolving evidence during the study period.^9^

Our patient had rapid clinical deterioration and had raised biomarkers of inflammation. We administered tofacitinib in addition to dexamethasone and enoxaparin. At the time of initiation of therapy, she had no evidence of active bacterial infection. Clinical improvement was evident after 48 hours or initiating the therapy with progressive improvement in the subsequent days. During her hospital stay, she did not develop any features of bacterial sepsis and new organ dysfunction. In parallel to the clinical improvement, her chest X-ray showed progressive improvement and her level of serum biomarkers progressively decreased.

The results of well-designed randomized trials, in selective patients with COVID-19, those with rapid deterioration in oxygenation, in hyperinflammatory state, and without the evidence of active bacterial infection, tofacitinib may be a valuable adjunct to corticosteroids and may potentially improve outcome.
